# Deciphering Pleiotropic Signatures of Regulatory SNPs in *Zea mays* L. Using Multi-Omics Data and Machine Learning Algorithms

**DOI:** 10.3390/ijms23095121

**Published:** 2022-05-04

**Authors:** Ataul Haleem, Selina Klees, Armin Otto Schmitt, Mehmet Gültas

**Affiliations:** 1 Breeding Informatics Group, Department of Animal Sciences, Georg-August University, Margarethe von Wrangell-Weg 7, 37075 Göttingen, Germany; ataul.haleem@uni-goettingen.de (A.H.); selina.klees@uni-goettingen.de (S.K.); armin.schmitt@uni-goettingen.de (A.O.S.); 2 Faculty of Agriculture, South Westphalia University of Applied Sciences, Lübecker Ring 2, 59494 Soest, Germany; 3 Center for Integrated Breeding Research (CiBreed), Georg-August University, Carl-Sprengel-Weg 1, 37075 Göttingen, Germany

**Keywords:** multi-omics, regulatory SNPs, incremental feature selection, random forest, markov clustering, hierarchical network model, gene expression profiles

## Abstract

Maize is one of the most widely grown cereals in the world. However, to address the challenges in maize breeding arising from climatic anomalies, there is a need for developing novel strategies to harness the power of multi-omics technologies. In this regard, pleiotropy is an important genetic phenomenon that can be utilized to simultaneously enhance multiple agronomic phenotypes in maize. In addition to pleiotropy, another aspect is the consideration of the regulatory SNPs (rSNPs) that are likely to have causal effects in phenotypic development. By incorporating both aspects in our study, we performed a systematic analysis based on multi-omics data to reveal the novel pleiotropic signatures of rSNPs in a global maize population. For this purpose, we first applied Random Forests and then Markov clustering algorithms to decipher the pleiotropic signatures of rSNPs, based on which hierarchical network models are constructed to elucidate the complex interplay among transcription factors, rSNPs, and phenotypes. The results obtained in our study could help to understand the genetic programs orchestrating multiple phenotypes and thus could provide novel breeding targets for the simultaneous improvement of several agronomic traits.

## 1. Introduction

Maize is an exceptional source of food, feed and fuel. It has become one of the most important cereal crops that feed the world by contributing 30% of the food calories for 4.5 billion people [1]. Over the past decade, maize production has increased remarkably by more than 1.16 million tons (FAOSTAT), but there is still a need for further yield increases to offset the food insecurity caused by the exponential increase in the world’s population. However, the dramatic fluctuations in global mean temperatures observed over the past decades pose a serious threat to sustainable crop production and demand better strategies of crop improvement.

To deal with above-mentioned issues, several biochemical, physiological and morphological traits of maize (such as grain yield and biomass) have been of individual importance in breeding research [2,3,4,5,6,7,8,9,10,11,12,13]. For this purpose, several association studies have been conducted for marker-assisted selection of superior genotypes by applying conventional genome-wide association studies (GWAS), which could provide essential information about the genetic architecture of genotype × phenotype interactions [14]. Despite the rich literature on GWAS and their application in plant breeding, they are still criticized for high false positive rates [15,16,17], requirement of large sample sizes for the detection of rare alleles [18] and missing heritability [19]. In order to overcome these limitations to certain extents, machine learning (ML) approaches, like Random Forests (RFs) or convolutional neural networks (CNNs), have been successfully applied to large genomic data sets, which employ non-parametric methods to decipher genotype × phenotype interactions [17,20,21,22,23,24,25,26]. Especially, recent studies [16,17,27,28] have demonstrated the utility of RF based-models for the analysis of a large number of loci and the identification of promising SNP candidates having strong associations with phenotypes. For example, Klees et al. [20] recently applied the RF approach to identify associations between the rapeseed oil content and regulatory SNPs (rSNPS), which are located in the promoter regions of genes and have a strong impact on the binding sites of transcription factors (TFs), thus affecting the development of phenotypes.

Another fundamental aspect of single-SNP-based studies (including rSNPs) is their utility to detect associations between a SNP and multiple phenotypes. Such type of associations of a single-SNP or a gene is referred to as pleiotropy [29,30,31], which, by definition, is a phenomenon of having a single genetic variant responsible for multiple phenotypes. Given the importance of pleiotropy, the aspect of investigating rSNPs could be quintessential for understanding the influence of variation in quantitative traits and their improvement against biotic and abiotic stresses. However, a limited number of studies have reported pleiotropic effects in maize [32,33,34,35,36,37,38,39,40]. The lack of such type of studies in maize can be compensated using modern multi-omics technologies, which have enabled the researchers to rapidly sequence large breeding populations and measure several phenotypes [41], facilitating pleiotropic studies. In particular, transcriptomics, proteomics, genomics, and phenomics are increasingly being used in plant sciences to gain a comprehensive understanding of complex genetic traits [20,42].

Recently, Liu et al. [43] generated a comprehensive multi-omics dataset of global maize germplasm, comprising genomic, transcriptomic and multiple phenotypic data of 368 maize inbred lines representing stiff-stock, non-stiff-stock, tropical, semi-tropical and mixed backgrounds [44] to identify genome-wide associations between individual SNPs and phenotypes [45,46,47,48,49].

Leveraging these multi-omics data, the main objectives of our study are to identify pleiotropic signatures of rSNPs in a systematic analysis and to construct hierarchical network models that could lead to new hypotheses to determine the crucial role of TFs controlling the development of different phenotypes in maize breeding research. Our analysis pipeline primarily consists of four distinct phases. In the first phase, rSNPs are identified, while in the second phase, the RF algorithm is used to identify relative importance of individual rSNP in phenotype associations [17,20]. Based on these association results, we identify pleiotropic rSNPs in the third phase using the Markov clustering algorithm [50], which is followed by the construction of hierarchical network models in the fourth phase that elucidate the complex interplay among TFs, rSNPs, and multiple phenotypes. Our findings demonstrate that systematic analysis of multi-omics data of global maize populations: (i) enables the identification of pleiotropic signatures of rSNPs along with their consequences on TF binding sites and; (ii) provides new insights into the genetic architecture and new breeding targets for the corresponding multiple phenotypes in maize.

## 2. Materials and Methods

**Figure 1:**
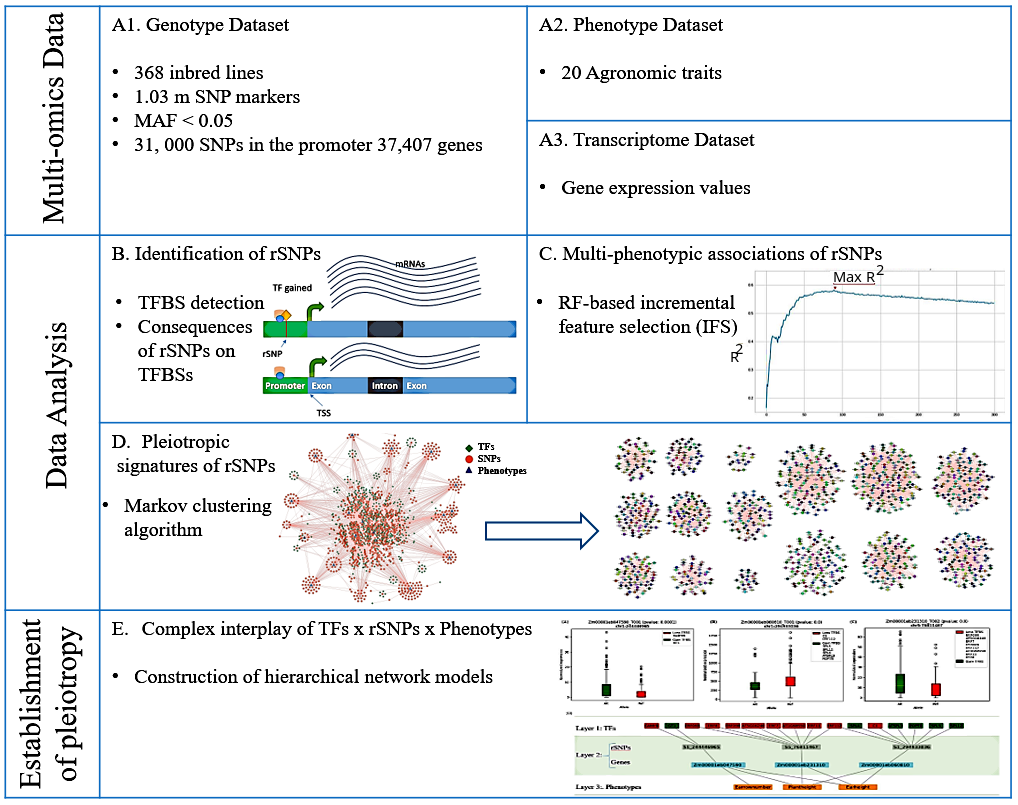
Overview of the analyses pipeline highlighting key machine learning algorithms for the identification of pleiotropic signatures of regulatory SNPs (rSNPs) to establish complex interplay of transcription factors (TFs), rSNPs and multiple phenotypes. The genotypic data (**A1**), consisting of 1.03 m SNP markers was filtered for MAF (<0.05) and 31,000 SNPs found within promoter regions of 37,407 maize genes were considered for association analysis with 20 quantitative agronomic traits (**A2**). RNA-seq (**A3**) dataset was utilized for the validation of pleiotropic rSNPs on the underlying gene expression. As of first step in the data analysis, rSNPs were identified (**B**) for their impact on the gain or loss of TFBSs, after which their association with multiple phenotypes was determined using random forest (RF) using the Boruta algorithm and incremental feature selection (IFS) technique (**C**). Pleiotropic signatures of rSNPs were then established by pruning weaker connections in the overall network into smaller non-overlapping fully connected clusters, using Markov clustering (MCL) algorithm (**D**) which provided the basis for the construction of hierarchical network models with three distinct layers modelling the complex interplay of TFs, rSNPs and multiple phenotype (**B**). Further, the boxplots show the impact of pleiotropic rSNPs at gene expression level as a function of gain or loss of TFBSs (**E**).

In this section, we describe the multi-omics dataset analyzed and the methods applied in this study. Our analysis framework is structured as shown in the Figure 1. In particular, we start with the preprocessing of multi-omics data of 368 maize inbred lines and their systematic analysis towards the identification of pleiotropic signatures of rSNPs. For this purpose, we first identified the rSNPs from the genotype dataset by applying the MATCH™ algorithm [51] together with a non-redundant plant position weight matrix (PWM) library obtained from the TRANSFAC database [52]. Second, the Random forest algorithm [53] was applied together with its specific feature selection wrapper, the Boruta algorithm [54], to assess the relative importance of each rSNP in terms of its involvement in the characterization of 20 agronomic phenotypes under study. This step was followed by the incremental feature selection (IFS) procedure [55,56] to find the optimal list of associated rSNPs for each phenotype. Next, using the Markov clustering algorithm (MCL) algorithm [50] the pleiotropic relationship signatures of rSNPs were uncovered, as suggested in [57]. Finally, we constructed hierarchical network models to elucidate the complex interplay among TFs, rSNPs, and multiple phenotypes by incorporating the corresponding transcriptome dataset to evaluate the importance of pleiotropic rSNPs. Detailed information on these analysis steps are given in Section 2.2 from Phase 1 to 4.

### 2.1. Multi-Omics Data

#### 2.1.1. Genotype Dataset

The genotypic data of 368 inbred lines was obtained from: (i) CIMMYT; (ii) the Germplasm Enhancement of Maize (GEM) project in the USA; (iii) temperate and tropical/subtropical breeding programs in China. The inbred lines represent non-stiff stock, stiff stock, mixed, tropical and semi-tropical backgrounds. The genotyping has been performed using the MaizeSNP50 BeadChip with 56,110 SNP markers [58] which was further incremented to 1.03 million SNP markers using deep RNA-Seq data [43]. Similar to previous studies [47,59], SNPs with an MAF <0.05 are discarded, and genomic coordinates for SNP markers are lifted over from reference genome V2 to V5 using CrossMap [60]. Consequently, after filtering, the genotype dataset comprises 31,934 SNPs for 368 maize lines which are located on the chromosomes 1 to 10 including 37,407 genes.

#### 2.1.2. Phenotype Dataset

The corresponding phenotypic data of the maize lines was collected in 2009 and 2010 in five different environments in China. The experimental units were completely randomized with a row length of 3 m, 11 plants per row, 25 cm spacing between plants, and 60 cm spacing between rows. The mean observed value of five randomly selected plants for 20 agronomic (quantitative) traits was taken, which additionally were converted into the best linear unbiased predictions (BLUPs) [40,48,49].

#### 2.1.3. Transcriptome Dataset

Paired-end deep RNA-seq data for the 368 inbred lines, generated by Liu et al. [43], was retrieved from the European Nucleotide Archive (ENA) browser (study accession: PRJNA208608). Raw sequencing data were adapter and quality trimmed using Trim Galore [61]. High-quality reads were then mapped to the *Zea mays* L. reference genome, V5 (available at https://download.maizegdb.org/Zm-B73-REFERENCE-NAM-5.0/Zm-B73-REFERENCE-NAM-5.0.fa.gz) (accessed on 16 April 2021), using STAR (v2.7.3a) [62]. Raw read counts for each transcript were obtained using HTSeq [63] and normalized using the median-of-ratios normalization method implemented in the R package DESeq2 [64].

### 2.2. Data Analysis

Our analysis framework consists of four phases to decipher the complex interplay among transcription factors (TFs), regulatory SNPs (rSNPs) and multiple phenotypes using the multi-omics dataset under study.

Phase 1: We identified rSNPs from the genotype dataset by applying our analysis pipeline introduced in [14], which consists of the following steps. First, considering the promoter region of genes (−500 bp to +100 bp relative to the transcription start site), all SNPs in these regions were selected. Second, we extracted the flanking sequence of each selected SNP, which covers ±25 bp relative to the SNP position. In total, each sequence is 51 bp long and the SNP is located in the central position. Then, two copies of the extracted sequences were constructed: while the first copy contains the reference allele at the SNP position, the second has the alternate allele. Next, by employing the MATCH™ program [51], we scanned each sequence to predict binding sites of transcription factors with their affinity scores ∈[0,1]. For the application of the MATCH™ program, we used a non-redundant plant position weight matrix (PWM) library obtained from the TRANSFAC database [52]. Finally, mainly focusing on the alterations of transcription factor binding sites (TFBSs) in the sequences of each SNP, we collected its potential consequence as: (i) ”Loss of TFBS”: only the sequence with the reference allele contains the TFBS of a specific transcription factor (TF), but the same TFBS is not found in the sequence with alternate allele; and (ii) “Gain of TFBS”: the TFBS can be found only in the sequence with the alternate allele; and (iii) “No Strong Effect”: indicating that the SNP consequence has either no effect or entails a slight change in binding affinity of TFs. As suggested in the previous studies [14,20,65], we consider in our following analysis a SNP as an rSNP if it leads to a “Gain of TFBS” or a “Loss of TFBS” for at least one TF.

Phase 2: Following the association analysis strategy described in [16,17,20], we used a Random Forest (RF)-based feature selection approach to assess the relative importance of each rSNP in predicting the response variable (phenotype) of interest. To this end, we applied the Boruta algorithm [54], a powerful wrapper designed specifically for the RF-based feature selection technique, to rank the importance of variables (in this case rSNPs). Consequently, by constructing multiple decision trees based on random subsets of features, the Boruta algorithm calculates an importance score for each rSNP and thus provides a ranking.

**Figure 2:**
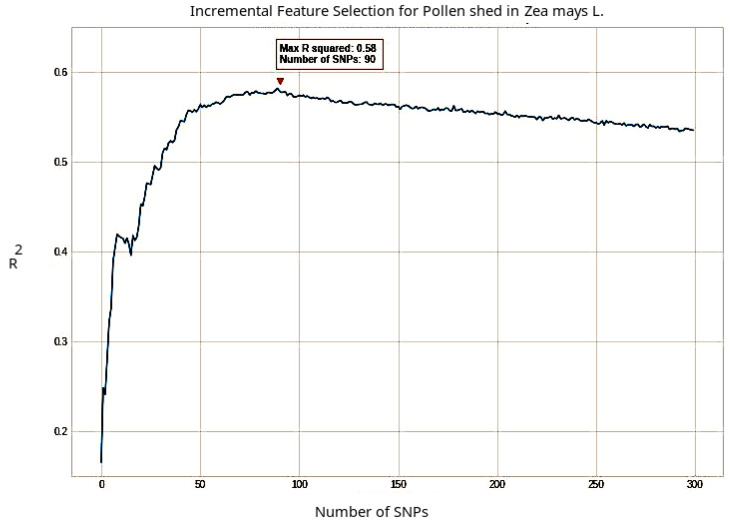
A plot to show the change of $R^{2}-$values versus the number of rSNPs in association with the phenotype pollen shed. The incremental feature selection (IFS) curves were drawn using the ranking of rSNPs. The $R^{2}-$ value reached a peak when considering the first 90 rSNPs. These rSNPs were used for the further analysis of this phenotype.

Using the ranked rSNPs determined by the Boruta algorithm, we further performed the incremental feature selection (IFS) procedure to retrieve the optimal list of features as suggested in [55,56]. During the IFS application, the rSNPs were incrementally added from higher to lower ranks in the ordered feature set, based on which an RF classifier was constructed. The predictive performance of RF was examined based on the $R^{2}$ values. This enabled us to determine the optimal numbers of associated rSNPs for a certain phenotype of interest (see Figure 2).

These analyses were repeated for each of the 20 phenotypes to identify the optimal numbers of the associated rSNPs, that are are given in Table 1.

Phase 3: To reveal the unique pleiotropic relationship signatures of rSNPs and thus decipher their complex interplay with TFs and with multiple phenotypes, we applied the Markov clustering algorithm (MCL). MCL is a very effective network-based clustering algorithm that detects distinct groups in a network by eliminating negligible connections (edges) based on their weights [50].

For the detection of pleiotropic relationships, Weighill et al. [57] have successfully applied the MCL algorithm by constructing a profile matrix which represents the SNP × phenotype associations. Following this idea and thus the main concept of MCL, we first created such a profile matrix M, where rows correspond to rSNPs determined by the IFS procedure and columns refer to names of both the phenotypes and TFs. The entry of M at position (i,j), $M_{ij}$ is defined as:

$$\begin{array}{c} M_{ij}=\left\{\begin{array}{cc} 1 & \;\mathrm{if}\;\;{\mathrm{rSNP}}_{i}\;\;\mathrm{is}\;\mathrm{associated}\;\mathrm{with}\;\mathrm{phenotype}\;j\\ 1 & \;\mathrm{if}\;\;\mathrm{the}\;\mathrm{consequence}\;\mathrm{of}\;{\mathrm{rSNP}}_{i}\;\;\mathrm{is}\;“\mathrm{Gain}”\;\mathrm{or}\;“\mathrm{Loss}”\;\mathrm{for}\;\mathrm{TF}\;j\\ 0 & \;\mathrm{otherwise}\; \end{array}\right. \end{array}$$

M was then converted into an rSNP association matrix, $A_{n\times n}$ (*n* is the number of rSNPs (rows) in M), using the Proportional Similarity Index [66]. The entry of A at a position (k,l) is calculated between the rSNPs (rows) *k* and *l* in M as:

$$A_{kl}=2\cdot \frac{\sum_{j}\min\left(M_{kj},\;M_{lj}\right)}{\sum_{j}\left(M_{kj}+M_{lj}\right)}$$

Next, we employed MCL [50] using the matrix A to cluster rSNPs in subgroups based on their similar relationship signatures. Consequently, each of the resulting clusters reflects a collection of rSNPs and their complex interplay with TFs and phenotypes, based on which we designed a hierarchical network model using three layers. These layers are: (i) TFs whose binding site is lost or gained due to the rSNPs; (ii) rSNPs located in the promoter of the genes; and (iii) phenotypes whose development is strongly connected to the expression level of the corresponding genes. In a final step, we carefully removed the rSNPs from the hierarchical network models if they were associated with only one phenotype for ensuring the pleiotropy in the clusters [57].

Phase 4: For the assessment of the consequences of pleiotropic rSNPs on TF-binding activities, which in turn affect the regulation of gene expression and thus the development of the phenotype, we evaluated their potential effects using the corresponding RNA-seq data. For this purpose, we focused only on genes with pleiotropic rSNPs in their promoters. By considering these genes, we divided the 368 maize lines into two groups for each pleiotropic rSNP: While the plants in the first group have the reference allele at the corresponding genomic position, the plants in the second group contain an alternate allele of the rSNP under study. As a result, we compared the gene expression values between those two groups using the Wilcoxon test in order to determine whether the consequence of a pleiotropic rSNP on a certain TF-binding activity (“Gain” or “Loss”) leads to a significant alteration in the expression of the corresponding gene.

In our following analysis, we further pruned the hierarchical network models by removing the rSNPs and corresponding TFs whose binding activities do not result in a significant change in the related gene expression values.

## 3. Results and Discussion

**Figure 3:**
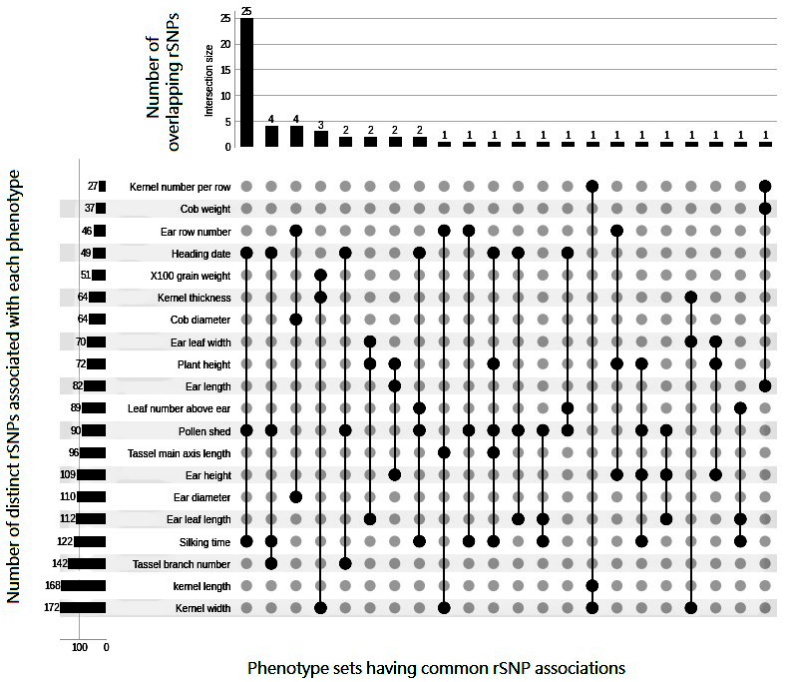
Number of associated rSNPs determined by the incremental feature selection (IFS) procedure for each phenotype and their overlap represented in matrix layouts using the UpSet technique [67]. Black circles in the matrix layout are related to the phenotypes that are part of the intersection. For the sake of clarity, not all intersections are displayed.

In this study, we systematically analyzed a multi-omics dataset of 368 maize inbred lines to decipher the complex interplay among TFs, rSNPs and multiple phenotypes. For this purpose, we first identified the rSNPs from genotype dataset and then applied the Boruta algorithm followed by IFS procedure to determine the rSNPs having a strong association with the phenotypes of interest. The number of associated rSNPs along with corresponding phenotypes is given in Table 1 and Figure 3. Next, considering the multi-phenotypic associations of the rSNPs as well as their consequences on TF binding, we employed the MCL algorithm to cluster the rSNPs, which is additionally used to construct hierarchical network models to elucidate the relationship signatures between TFs and rSNPs together with those between rSNPs and multiple phenotypes, indicating their pleiotropic functions.

### 3.1. Pleiotropic Association Signatures of rSNPs

The application of the MCL algorithm to the rSNPs determined by the IFS procedure (Table 1) results in eleven clusters that reveal the unique pleiotropic relationship signatures of rSNPs arising from their complex interplay with TFs and multiple associated phenotypes. A brief description of the clusters is given in Table 2 and additional information about rSNPs and genes are provided for each cluster in Supplementary File S1.

As shown in Table 2, the individual clusters are quite different regarding the numbers of pleiotropic rSNPs as well as their related phenotypes. The largest cluster contains ten genes (Zm00001eb354560, Zm00001eb403000, Zm00001eb328980, Zm00001eb137870, Zm00001eb366710, Zm00001eb190550, Zm00001eb364380, Zm00001eb156700, Zm00001eb337030, Zm00001eb397560) each associated with four phenotypes. Among these genes, Zm00001eb137870, which encodes the VIN3-like protein 1, is associated with heading date, silking time and pollen shed. Its homologue in arabdopsis, AT3G24440, is known as the Vernalization Insensitive 3-like 1 (VIL1) gene that regulates expression of the Flowering Locus C (FLC:floral repressors) and the Flowering Locus M (FLM) in response to vernalization. VIL1 and VIN3 (Vernalization Insensitive 3) are essential for epigenetic modification of the FLC and FLM loci [68,69]. The VIL1 gene acts upstream of many biological processes including administration and regulation of histone methylation, vernalization response and regulation of flower development. Additionally, it is a negative regulator of gene expression, which is achieved by its role in the positive regulation of histone H3-K27 methylation [70]. VIL1 is also involved in photoperiodism, flowering, vernalization response and response to cold [71]. Other genes in this cluster are known for their involvement in protein metabolism (Zm00001eb354560, Zm00001eb403000, Zm00001eb364380) and fat metabolism (Zm00001eb328980), however, the functionality of the remaining genes in this cluster is currently not known.

Another interesting instance of pleiotropy in action can be seen in Cluster-2, where most of the phenotypes are clustered together, representing a highly complex and coordinated developmental program of the disparate phenotypes in this cluster. The cluster contains genes belonging to protein metabolism (Zm00001eb131510, Zm00001eb288200), carbohydrate and fat metabolism (Zm00001eb372200, Zm00001eb418690), along with flowering time genes (Zm00001eb188340, Zm00001eb418690). Among these genes, Zm00001eb131510 encodes an intracellular protein transporter and is associated with ear leaf length and cob weight. This protein transporter is known for its role in exocytosis, golgi to plasma membrane transport and intracellular protein transport [70]. Its arabidopsis homologue, AT4G02350, is vital for pollen tube growth, pollen germination and acceptance in arabidopsis [72]. Such a gene may play an important role in the source-sink relationship between the developing maize kernels and the flag leaf for translocation of proteins. The arabidopsis homologue, AT1G66430, of the oil content related gene, Zm00001eb372200 is associated with ear row number and ear leaf height in this cluster and is also known for its role in carbohydrate biosynthetic, fatty acid biosynthetic, and fructose metabolic processes [73]. Several genes in this cluster indicates the co-regulation of floral transition and seed development in maize.

A closer look at Cluster-4 reveals that it contains six pleiotropic rSNPs found within five genes (Zm00001eb068110, Zm00001eb140090, Zm00001eb424640, Zm00001eb049750, Zm00001eb057150), associated with only ear traits (ear diameter, cob diameter and ear row number). The genes in this cluster are involved in riboflavin (Zm00001eb068110) [74] and the fatty acid metabolism (Zm00001eb049750) [47], protein relocalization to mitochondrion (Zm00001eb140090) [75] as well as acy carrier activity (Zm00001eb049750) [76]. The gene Zm00001eb049750 encoding the acyl carrier protein is known for its association with maize kernel oil contents [47], while the fax1 (AT3G57280) mutants, a homologue of Zm00001eb424640, are characterized by a decrease in biomass, plant height, stem thickness, reduced male sterility and defective pollen cell wall biosynthesis [77].

Among the smallest clusters are the Clusters 9-11, containing two pleiotropic rSNPs and two corresponding genes each. Additionally there are within the Cluster-10 another two kernel and ear development genes (Zm00001eb213840, Zm00001eb349520). The arabidopsis homologue (AT4G07960.1) of Zm00001eb213840 is known to encode XyG glucan synthase. Arabidopsis mutants of this gene have smaller rosettes and inflorescence stems, weak inflorescence stems and a reduced number of pollen tubes after pollination [78]. Moreover, the product of Zm00001eb349520 is known as a aspartic acid proteinase inhibitor. Today it is well known that high levels of aspartic acids are observed in maize cobs during early reproductive development [79]. Aspartic acids accumulate in kernels as N-assimilates, suggesting its role in kernel growth.

### 3.2. Construction of Hierarchical Network Models

**Figure 4:**
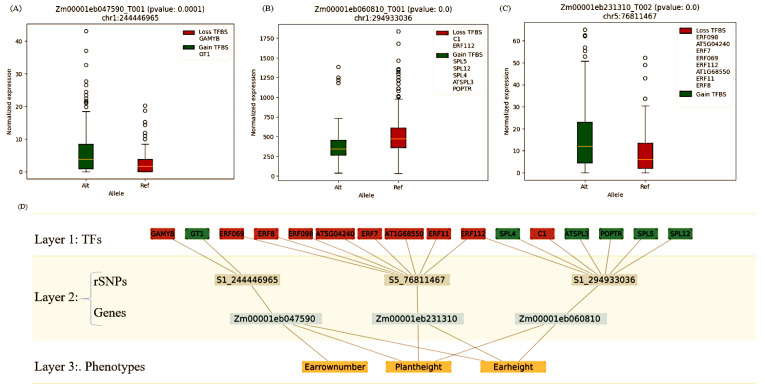
Hierarchical network model constructed using Cluster-7 to elucidate the complex interplay among TFs−rSNPs(genes)−Phenotypes. (**A**–**C**) show the significant changes in the gene expression values resulting from the consequences of pleiotropic rSNPs. (**D**) Hierarchical network model with three layers.

To further elaborate the complex interplay between pleiotropic rSNPs and TFs by assessing their potential impact on the expression level of the respective genes, we constructed a hierarchical network model for each cluster found by the MCL algorithm. These network models help explain the potential biological functions of TFs in regulating gene expression and, hence, assess the importance of individual pleiotropic rSNPs. They also provide with new hypotheses to advance our knowledge of why the consideration of the TFs could play a crucial role in maize breeding research to understand the genetic mechanisms underlying the development of different phenotypes. An example of our hierarchical network model is presented in Figure 4, showing the complex regulatory circuitry of Cluster-7 phenotypes. The phenotype set in this cluster represented by layer 3 (Figure 4D) comprises ear height, plant height and ear row number, whereas the layer 2 lists three associated rSNPs, three corresponding genes and their pleiotropic associations. Finally, in the first layer the TFs and the change in their binding activity is highlighted, showing loss of binding for ten TFs (in red) and gain of six (in green) TFs. In this cluster, a pleiotropic rSNP in Zm00001eb047590 (opf6 gene) results in the loss of a TFBS for GAMYB but in the gain for a GT1 binding site. The transcription factor GAMYB in arabidopsis is known for its role in Gibberelic acid (GA) signalling regarding floral transition [80,81], whereas the factor GT1 is vital to the regulation of stress tolerance in rice as well as response to light [82,83]. GT1 binds upstream of the light-responsive, rbcS-3A (RUBISCO) gene in peas, hence plays a crucial role in the regulation of photosynthesis [84]. The functional analysis of GT1 in arabidopsis shows its regulation in the target promoters that may have a repressive function in transcription activity [85]. Our findings indicate that the replacement of GAMYB with GT1 at the opf6 promoter (a transcription repressor gene) results in its significantly higher levels of gene expression (Figure 4A). This variation in gene expression may have an impact on the regulation of the GA pathway. For their role in flower development and flowering time [86], GA dynamic of the cells may contribute to the development of associated phenotypes (plant height, ear height and ear row number). Another pleiotropic rSNP in the bzip41 (Zm00001eb060810) promoter in this cluster results in loss of TFBSs for several SQUAMOSA-promoter binding protein-like TFs (SPL family TFs), whereas a TFBS is gained for C1 TF, resulting in significant change in gene expression (Figure 4B). C1 is known for regulating pigmentation in the aleurone layer of the maize kernels [87], whereas ERF112 is a potential negative regulator of the JA-responsive gene expression [88]. SPL proteins are a plant specific family of TFs and are known as potential candidates for the genetic improvement of agronomic traits due to their role in the physiological and reproductive development of plants [89]. For example, SPL4 is required for developmental transition and plays an important role in the determination of flowering time [90,91], whereas SPL12 is known to be expressed during plant development [92] and is responsive to abscisic acid biosynthesis in heat stress [93]. The association of this rSNP and recruitment of respective TFs is in line with the development of associated phenotypes. The pleiotropic rSNP (chr5:76811467) results in the loss of TFBSs for several ethylene responsive transcription factors (ERFs), which significantly increases the gene expression (Figure 4C). ERF TFs in this cluster mainly act as inhibitor of transcription [88,94], hence loss of their TFBS is translated as higher promoter activity on this gene, which may further contribute to the development of the associated phenotypes. Our results also show the importance of hierarchical network models in explaining the impact of pleiotropic rSNP on the expression of corresponding genes, which may directly influence all the associated phenotypes. TF−rSNPs(gene)−Phenotype network models for all other clusters are given in the Supplementary File S2.

## 4. Conclusions

Pleiotropy and rSNPs are the two main concepts in the field of genetics by providing novel targets for the acceleration of plant breeding strategies. Therefore we considered in this study both of these concepts together and established hierarchical network models for elucidating the complex interplay among TFs−rSNPs−Phenotypes using multi-omics data. Our results show that most of the identified TFs and genes play essential roles for the development of multiple phenotypes. Our findings further suggest common genetic mechanisms underlying several interrelated phenotypes found in Clusters-1 or -8 as well as disparate phenotypes, like plant height and kernel traits, found as in Clusters-2 or -5. To the best of our knowledge, by mainly focusing on the important role of rSNPs and their consequences on TFs, this is the first study which provides the pleiotropic relations of several agronomically important phenotypes of maize. The outcomes of our analysis could be highly relevant for the understanding of the genetic programs governing the development of multiple phenotypes. Therefore, further molecular biology progress is needed not only to assess the potential role of these rSNP candidates, but also to gain a deeper insight into the genetic mechanisms underlying biological processes in maize.

## 5. Future Directions

Unraveling the genetic architecture of complex traits is a key component for improving plants against biotic and biotic stresses. In this context, plant breeding strategies focus on developing QTL maps for different types of stresses, accounting for linkage disequilibrium and high false positive rate, standardizing genome-wide polygenetic scores [95,96], and incorporating epistatic effects into genome-wide association studies to explain missing heritability and pleiotropy using traditional marker-assisted selection (MAS) and genomic prediction (GS) strategies as well as ML approaches [97,98,99]. As the desired and undesired traits could share a pleiotropic relationship, consideration of pleiotropy is essential to direct breeding programs. Additionally consideration of pleiotropic signatures of rSNPs in the analysis of large genomic datasets with regarding MAS and GS, polygenetic scores, and polygenetic biotic interactions can impact the outcome of the analysis. We suggest that the incorporation of pleiotropic effects of rSNPs in the analysis of genomic data could improve outcome of MAS as well as GS studies.

## Figures and Tables

**Table 1:** Phenotypes and the optimal numbers of their associated rSNPs determined by incremental feature selection (IFS) procedure.

Phenotype	Max $R^{2}$	#rSNPs
Leaf number above ear	0.490740	89
Ear leaf width	0.484029	70
Cob diameter	0.445720	64
Ear height	0.509523	109
Kernel width	0.418115	172
Ear leaf length	0.553292	112
Tassel main axis length	0.498562	96
Pollen shed	0.581765	90
Heading date	0.537987	49
Ear length	0.434011	82
Silking time	0.506520	122
Ear diameter	0.481445	110
Cob weight	0.460850	37
X100 grain weight	0.389332	51
Tassel branch number	0.507112	142
Ear row number	0.491663	46
Kernel number per row	0.350717	27
Plant height	0.532837	72
kernel length	0.580691	168
Kernel thickness	0.437589	64

**Table 2:** Result of Markov clustering algorithm (MCL) including the numbers of rSNPs together with their related genes and their associated multiple phenotypes.

Cluster	Numbers of Pleiotropic		Phenotypes
	rSNPs	Genes	
Cluster-1	15	10	Heading date, Pollen shed, Silking time and Ear height
Cluster-2	9	7	Cob weight, Heading date, Pollen shed, Tassel main axis length, Ear leaf length, Plant height, Ear leaf width, Ear row number and Ear height
Cluster-3	7	6	Kernel length, Kernel thickness, Kernel number per row, Ear diameter and X100 grain weight
Cluster-4	6	5	Ear diameter, Cob diameter and Ear row number
Cluster-5	6	4	Heading date, Pollen shed, Silking time, Ear height and Tassel branch number
Cluster-6	5	3	Ear diameter, Cob diameter and Ear row number
Cluster-7	3	3	Ear height, Plant height and Ear row number
Cluster-8	3	3	Kernel width, Kernel length, Kernel thickness and X100 grain weight
Cluster-9	2	2	Ear leaf length, Leaf number above ear, Kernel length
Cluster-10	2	2	Ear length and Kernel number per row
Cluster-11	2	2	Ear diameter, Tassel main axis length and Cob weight

## Supplementary Materials

The following supporting information can be downloaded at: https://www.mdpi.com/article/10.3390/ijms23095121/s1.
