# Supplementary material for: Deciphering Pleiotropic Signatures of Regulatory SNPs in Zea mays L. Using Multi-Omics Data and Machine Learning Algorithms
Source: Int J Mol Sci. 2022 May 4;23(9):5121. doi: 10.3390/ijms23095121 (PMC9100765; doi:10.3390/ijms23095121)
Supplement: Supplementary file 1 [file ijms-23-05121-s001.zip › supplementaryFile2.pdf]

Supplementary Figure S1: Hierarchical network model for Cluster-1

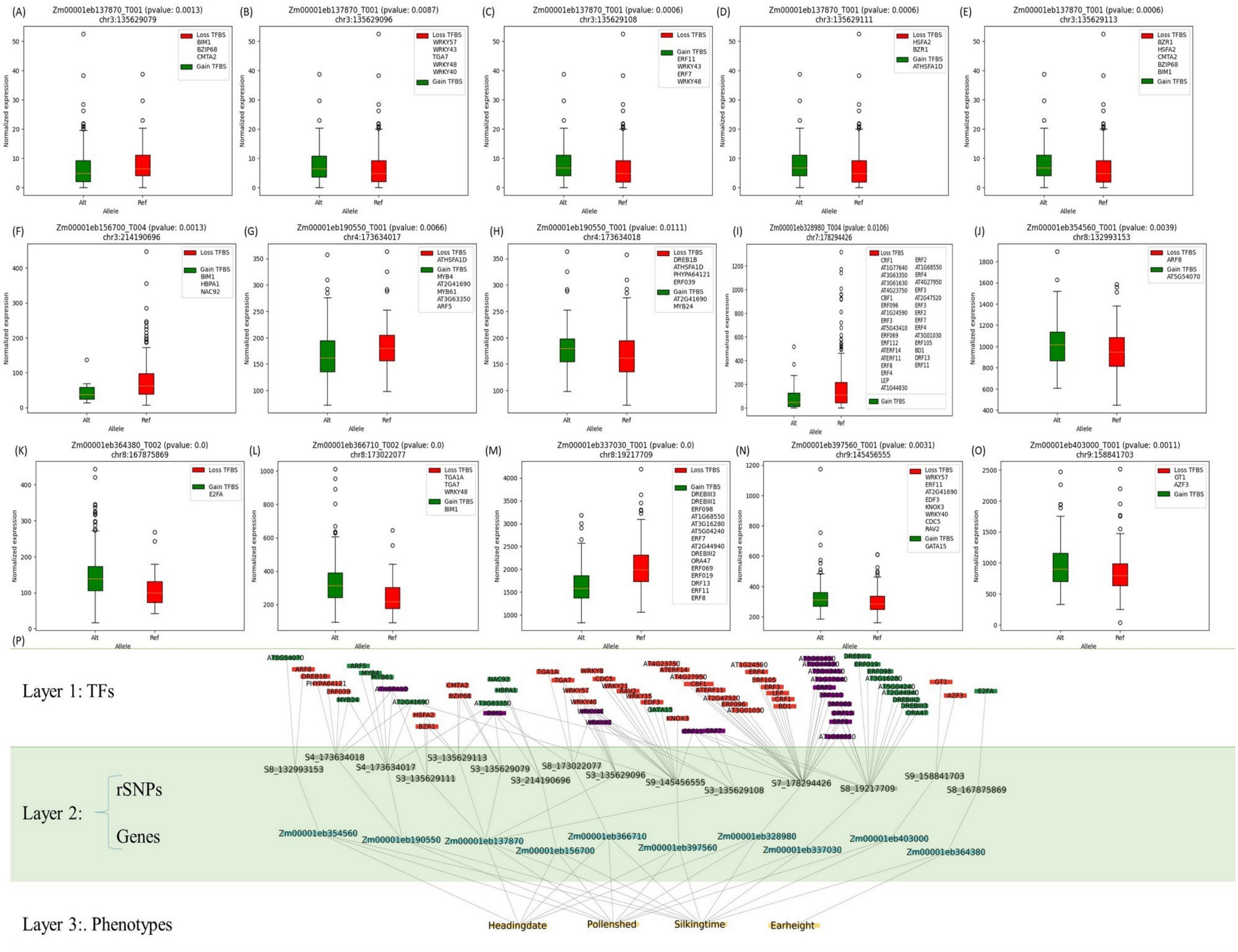


*Supplementary figure S1: Hierarchical network model constructed using Cluster-1 to elucidate the complex interplay among TFs−rSNPs(genes)−Phenotypes. (A–O) show the significant changes in the gene expression values resulting from the consequences of pleiotropic rSNPs. (P) Hierarchical network model with three layers. TFs are marked green and red for gain and loss of TFBSs, respectively, whereas purple color indicates that the TFBS for a particular TF is gained for one but lost for the other corresponding gene due to rSNPs in their promoter regions.*

Supplementary Figure S2: Hierarchical network model for Cluster-2

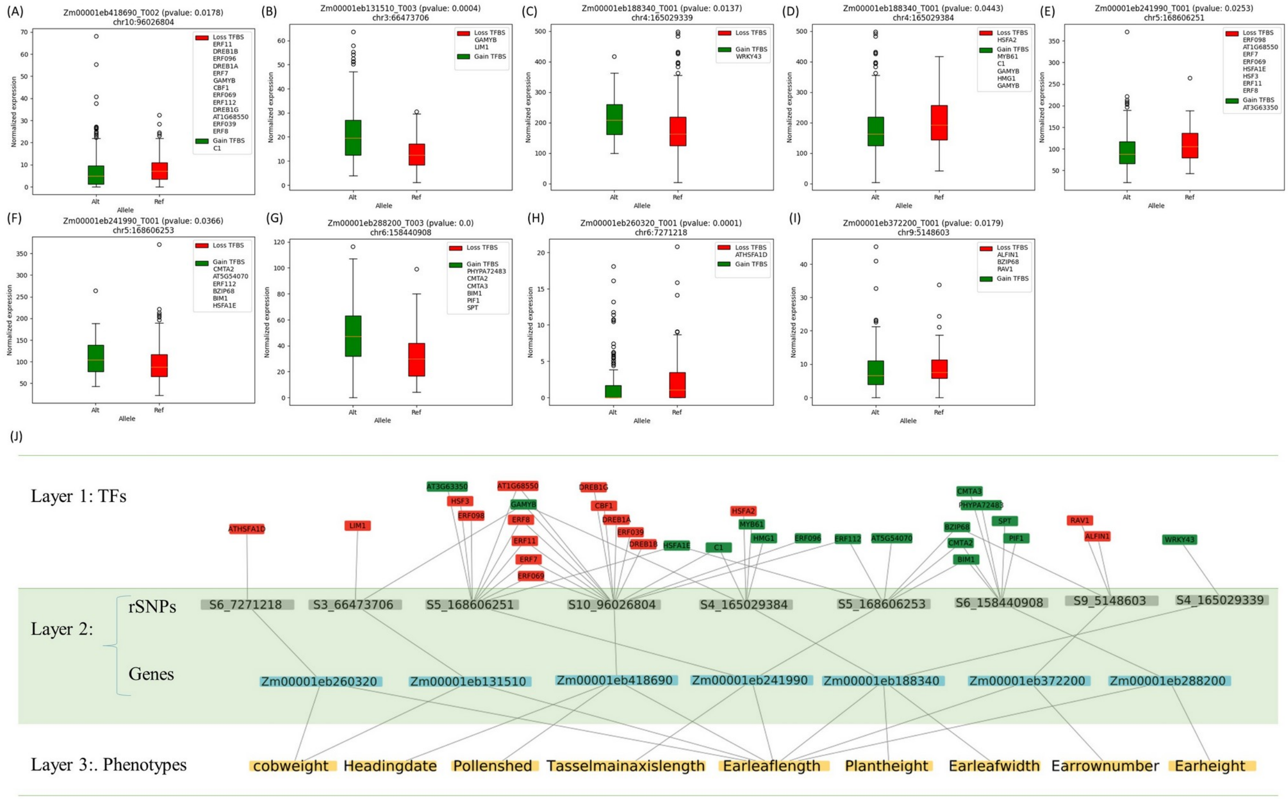


*Supplementary figure S2: Hierarchical network model constructed using Cluster-2 to elucidate the complex interplay among TFs−rSNPs(genes)−Phenotypes. (A–I) show the significant changes in the gene expression values resulting from the consequences of pleiotropic rSNPs. (J) Hierarchical network model with three layers. TFs are marked green and red for gain and loss of TFBSs respectively.*

# Supplementary Figure S3: Hierarchical network model for Cluster-3

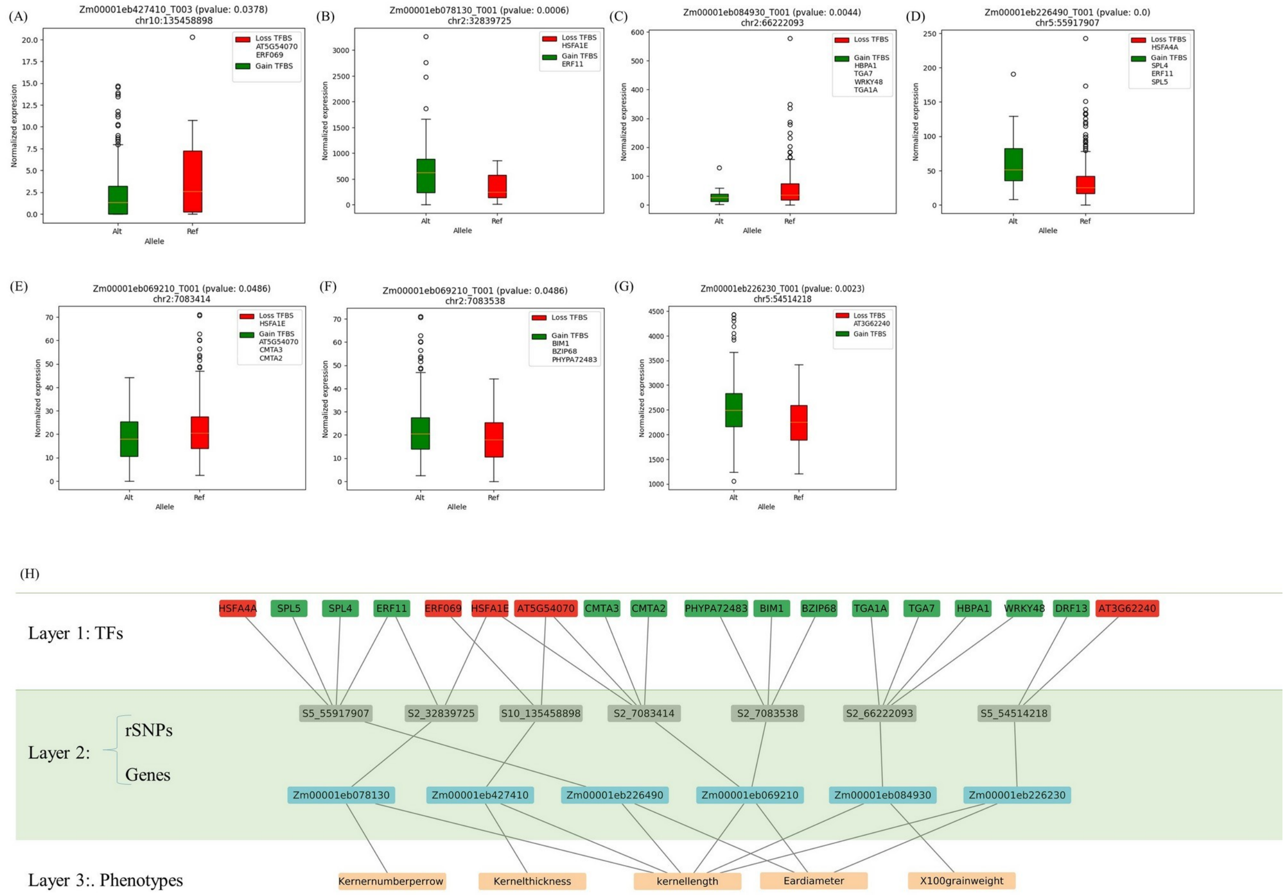


*Supplementary figure S3: Hierarchical network model constructed using Cluster-2 to elucidate the complex interplay among TFs−rSNPs(genes)−Phenotypes. (A–G) show the significant changes in the gene expression values resulting from the consequences of pleiotropic rSNPs. (H) Hierarchical network model with three layers. TFs  are marked green and red for gain and loss of TFBSs respectively.*

Supplementary Figure S4: Hierarchical network model for Cluster-4

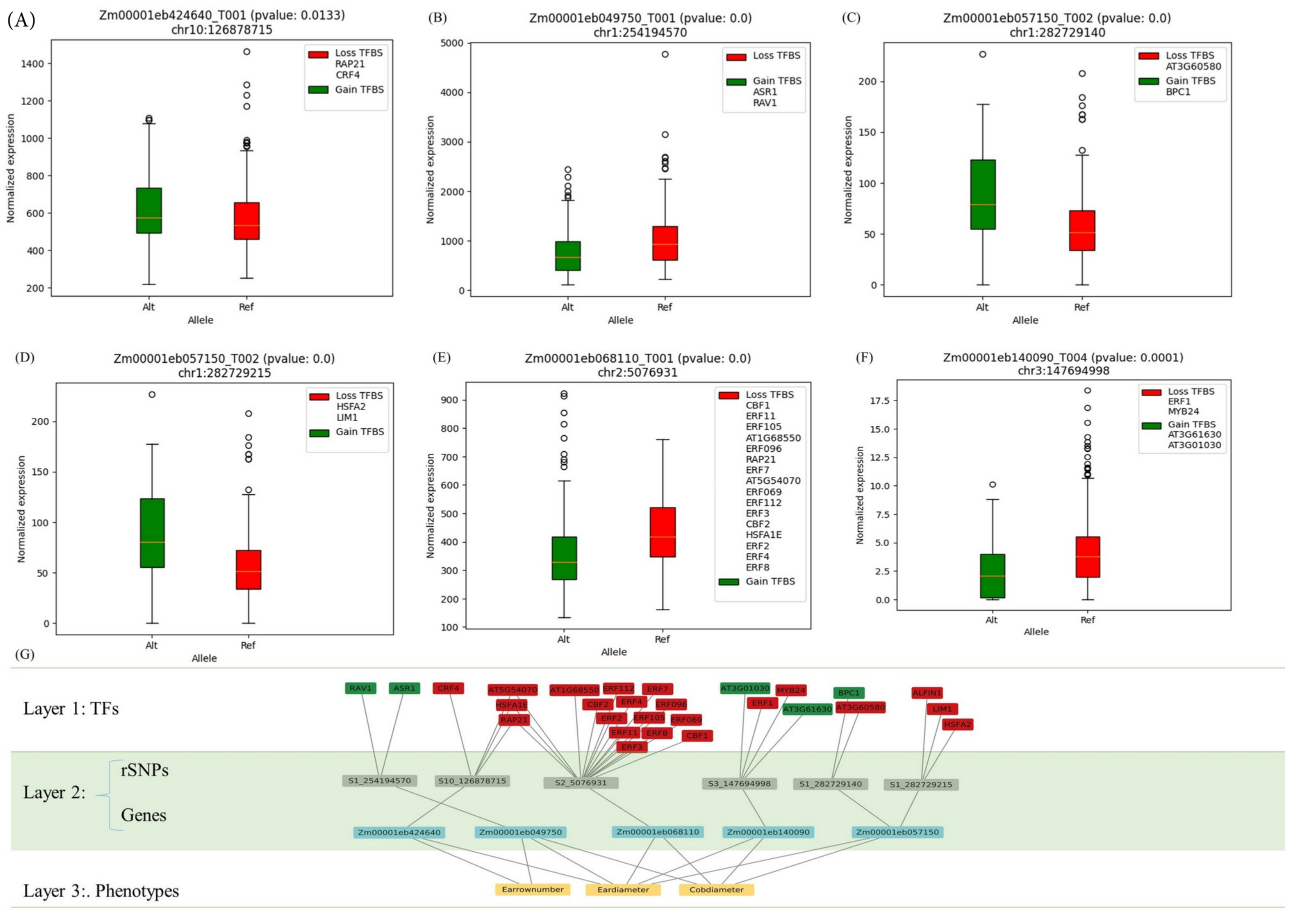


*Supplementary figure S4: Hierarchical network model constructed using Cluster-2 to elucidate the complex interplay among TFs−rSNPs(genes)−Phenotypes. (A–F) show the significant changes in the gene expression values resulting from the consequences of pleiotropic rSNPs. (G) Hierarchical network model with three layers. TFs  are marked green and red for gain and loss of TFBSs respectively.*

Supplementary Figure S5: Hierarchical network model for Cluster-5

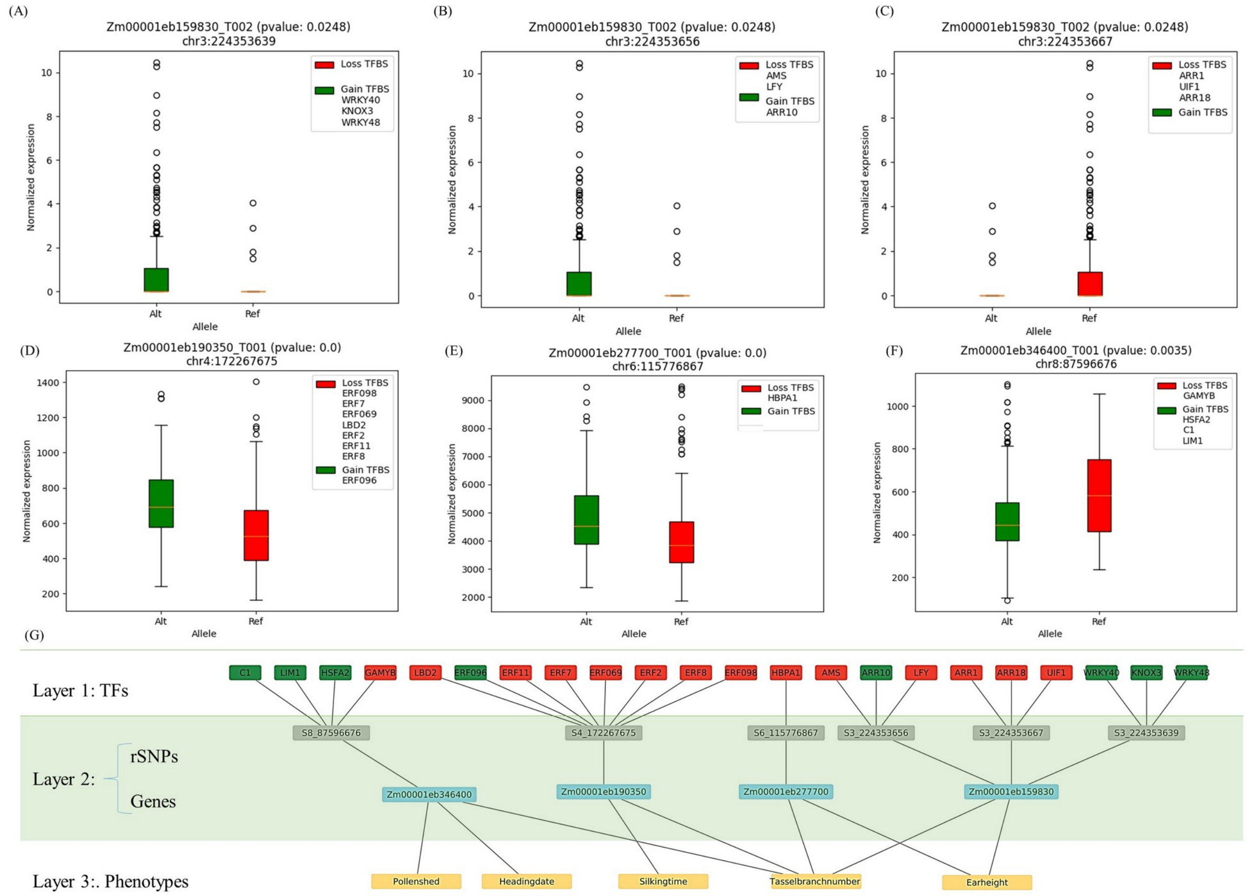


*Supplementary figure S5: Hierarchical network model constructed using Cluster-2 to elucidate the complex interplay among TFs−rSNPs(genes)−Phenotypes. (A–F) show the significant changes in the gene expression values resulting from the consequences of pleiotropic rSNPs. (G) Hierarchical network model with three layers. TFs  are marked green and red for gain and loss of TFBSs respectively.*

Supplementary Figure S6: Hierarchical network model for Cluster-6

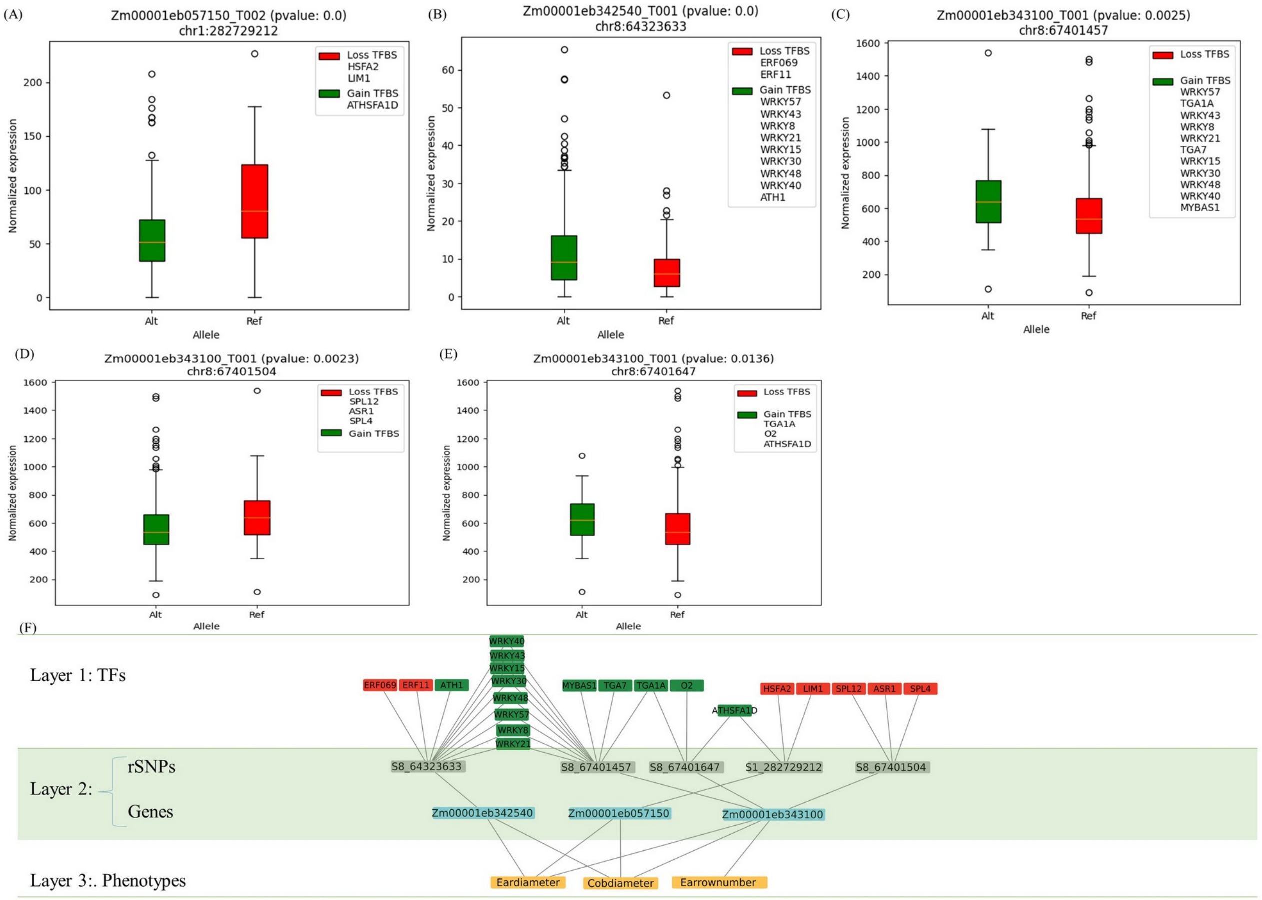


*Supplementary figure S6: Hierarchical network model constructed using Cluster-2 to elucidate the complex interplay among TFs−rSNPs(genes)−Phenotypes. (A–E) show the significant changes in the gene expression values resulting from the consequences of pleiotropic rSNPs. (F) Hierarchical network model with three layers. TFs are marked green and red for gain and loss of TFBSs respectively.*

Supplementary Figure S7: Hierarchical network model for Cluster-7

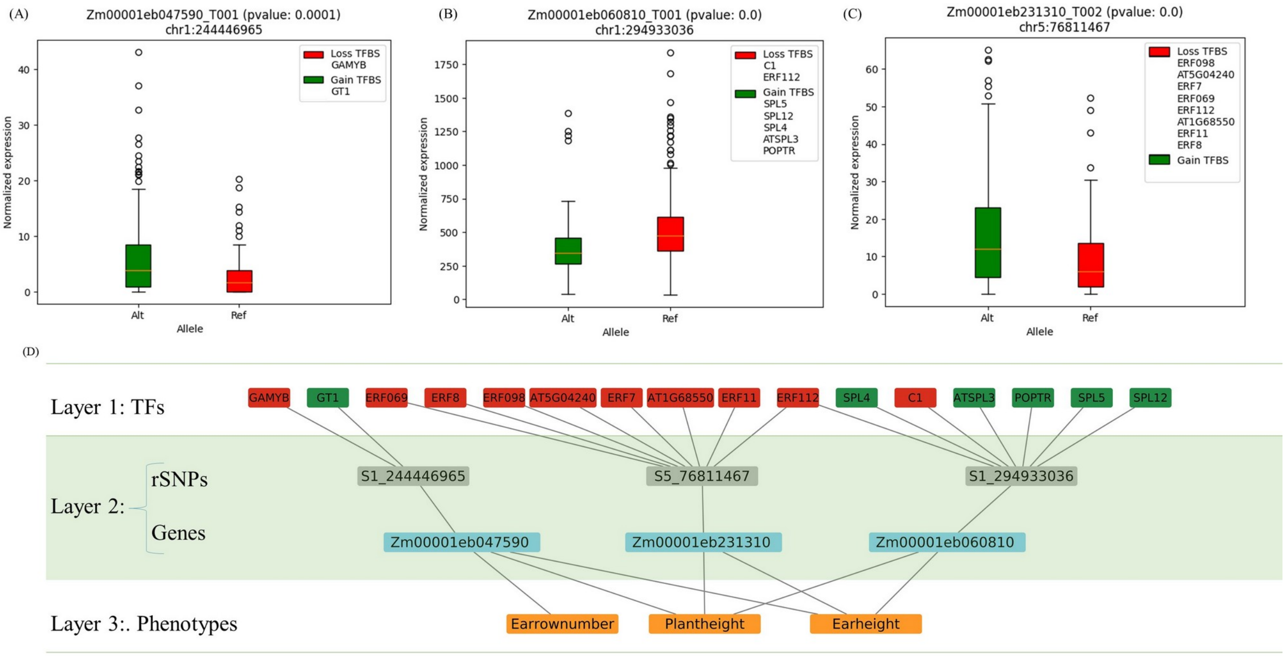


*Supplementary figure S7: Hierarchical network model constructed using Cluster-2 to elucidate the complex interplay among TFs−rSNPs(genes)−Phenotypes. (A–C) show the significant changes in the gene expression values resulting from the consequences of pleiotropic rSNPs. (D) Hierarchical network model with three layers. TFs  are marked green and red for gain and loss of TFBSs respectively.*

Supplementary Figure S8: Hierarchical network model for Cluster-8

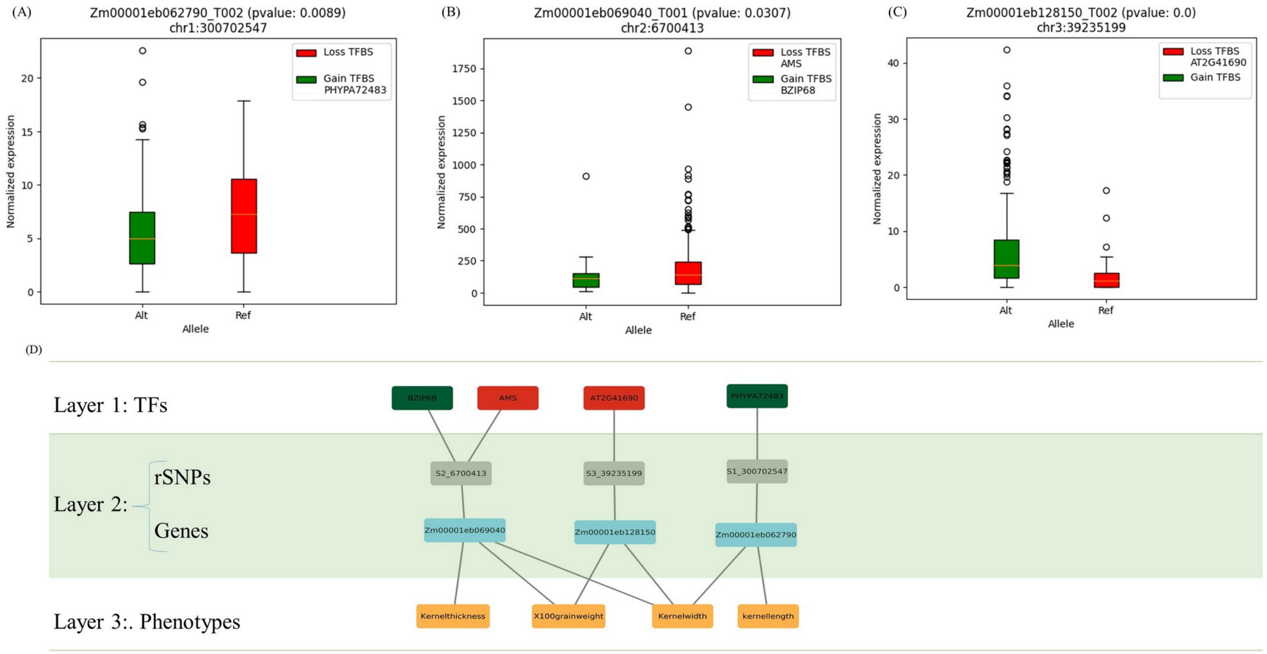


*Supplementary figure S8 : Hierarchical network model constructed using Cluster-2 to elucidate the complex interplay among TFs−rSNPs(genes)−Phenotypes. (A–C) show the significant changes in the gene expression values resulting from the consequences of pleiotropic rSNPs. (D) Hierarchical network model with three layers. TFs  are marked green and red for gain and loss of TFBSs respectively.*

Supplementary Figure S9: Hierarchical network model for Cluster-9

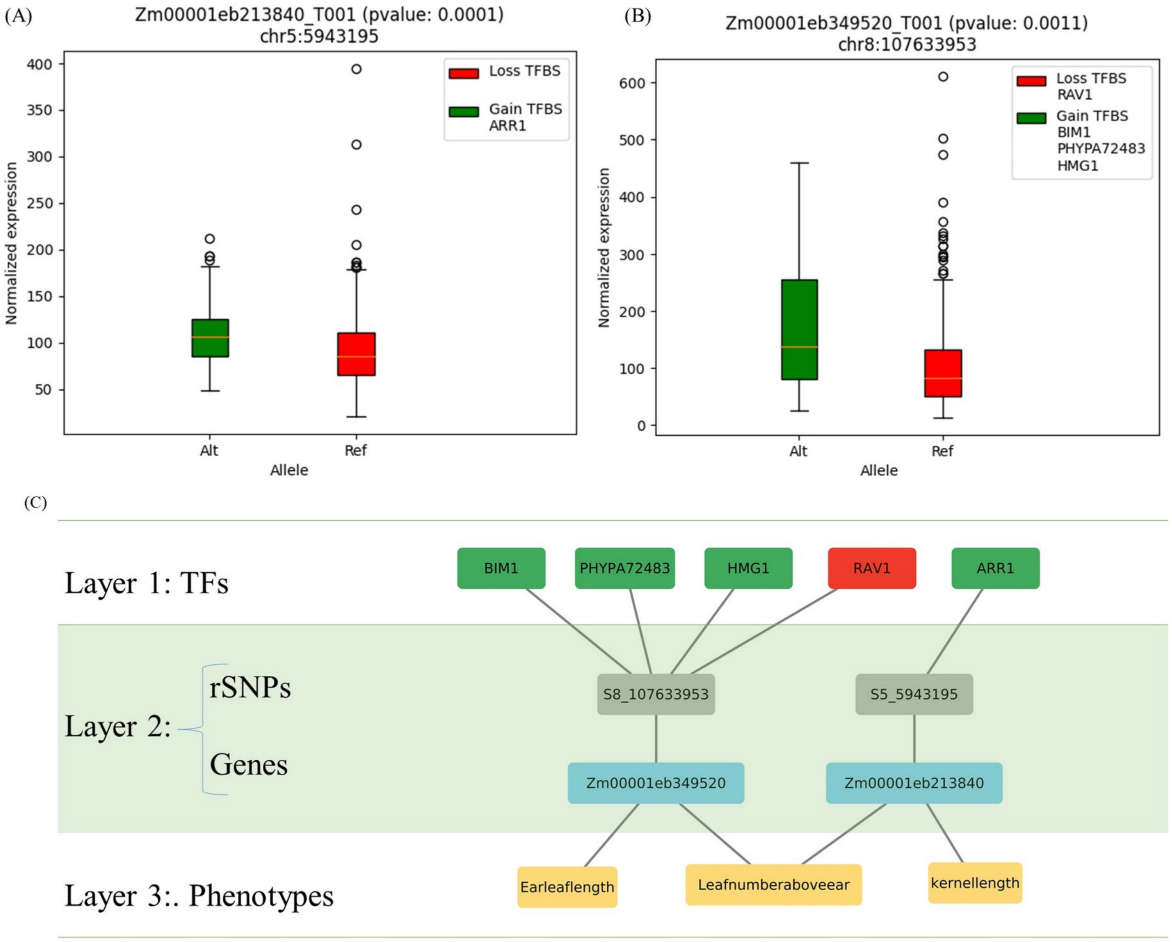


*Supplementary figure S9: Hierarchical network model constructed using Cluster-2 to elucidate the complex interplay among TFs−rSNPs(genes)−Phenotypes. (A and B) show the significant changes in the gene expression values resulting from the consequences of pleiotropic rSNPs. (C) Hierarchical network model with three layers. TFs are marked green and red for gain and loss of TFBSs respectively.*

Supplementary Figure S10: Hierarchical network model for Cluster-10

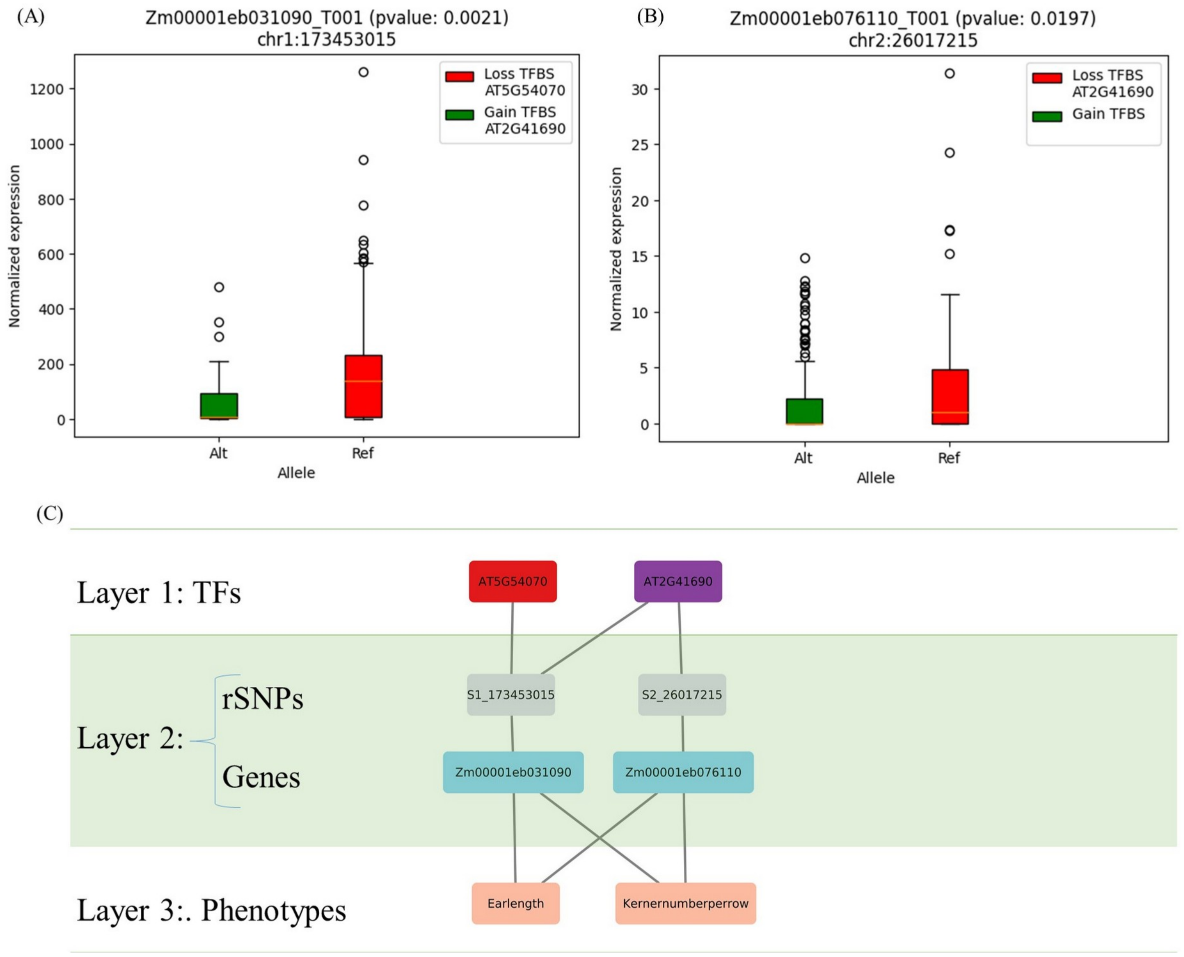


*Supplementary figure S10: Hierarchical network model constructed using Cluster-2 to elucidate the complex interplay among TFs−rSNPs(genes)−Phenotypes. (A and B) show the significant changes in the gene expression values resulting from the consequences of pleiotropic rSNPs. (C) Hierarchical network model with three layers. TFs  are marked green and red for gain and loss of TFBSs respectively.*

Supplementary Figure S11: Hierarchical network model for Cluster-11

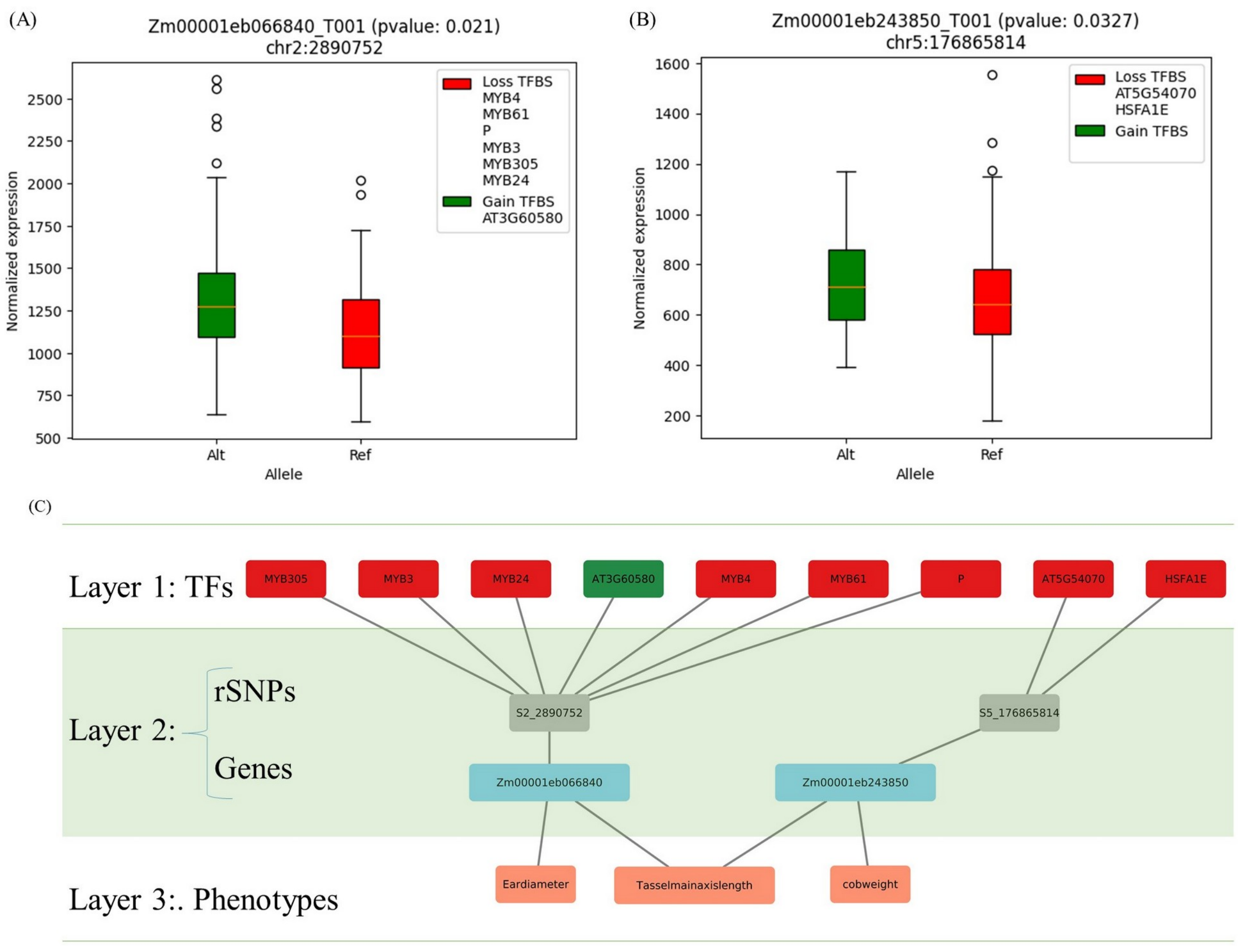


*Supplementary figure S11: Hierarchical network model constructed using Cluster-2 to elucidate the complex interplay among TFs−rSNPs(genes)−Phenotypes. (A and B) show the significant changes in the gene expression values resulting from the consequences of pleiotropic rSNPs. (C) Hierarchical network model with three layers. TFs  are marked green and red for gain and loss of TFBSs respectively.*
